# Smartphone Biosensors for Non-Invasive Drug Monitoring in Saliva

**DOI:** 10.3390/bios15030163

**Published:** 2025-03-04

**Authors:** Atheer Awad, Lucía Rodríguez-Pombo, Paula Esteiro Simón, André Campos Álvarez, Carmen Alvarez-Lorenzo, Abdul W. Basit, Alvaro Goyanes

**Affiliations:** 1 Department of Clinical, Pharmaceutical and Biological Sciences, University of Hertfordshire, College Lane, Hatfield AL10 9AB, UK; a.awad@herts.ac.uk; 2 Department of Pharmaceutics, UCL School of Pharmacy, University College London, 29-39 Brunswick Square, London WC1N 1AX, UK; 3 Departamento de Farmacología, Farmacia y Tecnología Farmacéutica, I+D Farma (GI-1645), Facultad de Farmacia, Instituto de Materiales (iMATUS) and Health Research Institute of Santiago de Compostela (IDIS), Universidade de Santiago de Compostela, 15782 Santiago de Compostela, Spain; lucia.rodriguez.pombo@rai.usc.es (L.R.-P.); paula.esteiro@uvigo.es (P.E.S.); carmen.alvarez.lorenzo@usc.es (C.A.-L.); 4 FABRX Ltd., Henwood House, Henwood, Ashford TN24 8DH, UK; andre.campos@fabrx.co.uk; 5 FABRX Artificial Intelligence, Carretera de Escairón, 14, Currelos, 27543 O Saviñao, Spain

**Keywords:** colorimetric and electrochemical biosensing, RGB profiling, salivary excretions and biological fluids, patient-centric diagnostics, smartphone-driven analysis, mobile phone biosensor app, digital analysis, portable point-of-care diagnostics, digitised medication analysis, remote drug monitoring

## Abstract

In recent years, biosensors have emerged as a promising solution for therapeutic drug monitoring (TDM), offering automated systems for rapid chemical analyses with minimal pre-treatment requirements. The use of saliva as a biological sample matrix offers distinct advantages, including non-invasiveness, cost-effectiveness, and reduced susceptibility to fluid intake fluctuations compared to alternative methods. The aim of this study was to explore and compare two types of low-cost biosensors, namely, the colourimetric and electrochemical methodologies, for quantifying paracetamol (acetaminophen) concentrations within artificial saliva using the MediMeter app, which has been specifically developed for this application. The research encompassed extensive optimisations and methodological refinements to ensure the results were robust and reliable. Material selection and parameter adjustments minimised external interferences, enhancing measurement accuracy. Both the colourimetric and electrochemical methods successfully determined paracetamol concentrations within the therapeutic range of 0.01–0.05 mg/mL (R^2^ = 0.939 for colourimetric and R^2^ = 0.988 for electrochemical). While both techniques offered different advantages, the electrochemical approach showed better precision (i.e., standard deviation of response = 0.1041 mg/mL) and speed (i.e., ~1 min). These findings highlight the potential use of biosensors in drug concentration determination, with the choice of technology dependent on specific application requirements. The development of an affordable, non-invasive and rapid biosensing system holds promise for remote drug concentration monitoring, reducing the need for invasive approaches and hospital visits. Future research could extend these methodologies to practical clinical applications, encouraging the use of TDM for enhanced precision, accessibility, and real-time patient-centric care.

## 1. Introduction

Accurately determining drug concentrations in the blood is crucial for various medical applications, including evaluating therapeutic profiles, assessing drug interactions, monitoring treatment cessation, and ensuring patient adherence [1]. Therapeutic drug monitoring (TDM) involves quantifying the drug in blood in order to optimise its effectiveness while avoiding toxicity, especially for a drug with a narrow therapeutic index (NTI).

High-performance liquid chromatography (HPLC) coupled with ultraviolet (UV) detection or mass spectrometry (LC-MS), and immunoassay techniques like fluorescence polarisation immunoassay (FPIA) and enzyme-linked immunosorbent assay (ELISA), are among the most widely used analytical methods for the estimation of drug concentrations, especially during clinical trials [2,3,4,5]. However, these traditional methods have limitations, including the need for blood sample collection by trained personnel in hospitals, patient discomfort and the use of expensive reagents [6,7,8,9]. Moreover, they lack portability and ease of interpretation, and therefore have seldom been used in point-of-care (PoC) diagnostics to provide ready-to-hand, quick feedback on medical tests to a patient and facilitate faster therapeutic interventions.

To overcome these limitations, biosensors present an ideal solution for TDM. They offer an automated system that provides rapid chemical analyses with minimal pre-treatment and delivers results within minutes [10]. Moreover, biosensors have the ability to analyse a range of matrices. As an example, wearable sensors are being explored to quantify different drugs or biomarkers in biological fluids, including saliva [11], sweat [12,13] and even tears [14]. These biosensors consist of biocatalysts within an analytical device that converts a biological element into a detectable electrochemical signal [15], with the biological element being based on enzymes, tissues, immune components, deoxyribonucleic acid (DNA), and others. The abundance of mobile phones worldwide, standing at 5.11 billion at present [16], with over half of these devices being smartphones, opens up the possibility of coupling smartphone technology with biosensors to perform quick, robust, and simple bioassays for PoC diagnostics [17]. This integration accelerates the development of user-friendly analytical devices for use at home or in remote settings, enabling the qualitative and quantitative determination of analytes using different indicators [18].

Traditionally, biological fluids such as blood and urine have been employed for drug monitoring, but come with significant disadvantages, including invasiveness, risk of infection transmission, sensitivity to hydration status, and higher resource demands [19]. In contrast, saliva offers several advantages [20,21,22,23], being non-invasive, cost-effective, and less affected by fluid intake variations [24]. Moreover, certain drugs, like paracetamol, exhibit a strong correlation between saliva and blood concentrations, establishing saliva as a promising medium for drug level monitoring, providing a patient-centric, affordable, and reliable alternative to conventional fluids [25]. As such, saliva is being studied as a viable biological fluid for the quantification of a diverse range of drugs, including substances associated with abuse and doping [26,27].

Among the various biosensor methods, colourimetry has gained attention for its potential use in PoC diagnostics. Colourimetric diagnostics based on aqueous solution [28] or paper [29,30,31,32,33] have been explored for various applications, including medical [34,35] and environmental [36] analyses. Interestingly, colourimetric sensors injected into the dermis in the form of tattoos have also been explored for the determination of metabolites (e.g., pH, glucose, and albumin) in an ex vivo skin tissue model [37]. However, their lack of specificity has hindered their widespread adoption in clinical and home settings. To address these challenges, the combination of smartphone technology and biosensors provides a more reliable method for analyte detection. By utilising red, green, and blue (RGB) profiling, this system translates increasing colour intensities to corresponding analyte concentrations [37,38]. Therefore, a smartphone-based colourimetric method could modernise PoC diagnostics, including the quantification of drugs like paracetamol, which has seen an increase in overdose cases in recent years [39].

An electrochemical technique performed with a smartphone is another alternative to colourimetry. This method not only improves accuracy and precision, but is also simple to use and portable, enabling the selective detection of compounds. However, it is important to note that a potential limitation of these devices can be their cost [40,41]. In recent years, there has been a growing interest in the development of new, more affordable, and accessible electrochemical devices [42]. Amongst these, the KickStat stands out as a potentiostat device combining extended electrochemical capabilities with a compact form. What truly distinguishes the KickStat is its cost-effectiveness, rendering it economically accessible [43]. Another notable attribute of the KickStat is its low operational voltage and high resolution, making it the device of choice when compared to top-tier benchtop potentiostats [43].

This study focused on the creation of a rapid, portable and user-friendly biosensing approach using the proprietary prototype smartphone app ‘MediMeter’, dedicated to the detection of paracetamol levels in saliva. MediMeter was specially developed for this study and allows the measuring of drug concentrations within a few minutes using different types of biosensing approaches. Paracetamol is an analgesic and antipyretic drug commonly used worldwide, but it is prone to overdose due to the perception of its safety [44]. For this reason, patients would benefit from the use of a portable biosensor that provides a user-friendly and cost-effective solution for paracetamol monitoring, enabling the early detection of potential overdosing [45]. Two low-cost analytical techniques, namely, the colourimetric and electrochemical methods, have been employed and compared to quantify paracetamol concentrations within artificial saliva. To achieve reliable results in both methods, various parameters were fine-tuned to mitigate external interferences and augment measurement precision.

## 2. Results and Discussion

In this work, the paracetamol concentration in artificial saliva was effectively measured using two biosensing methodologies (colourimetric and electrochemical) combined with the MediMeter mobile app. The measurements spanned a concentration range from 0.01 mg/mL to 0.05 mg/mL. Saliva samples were selected as an alternative to blood samples due to their non-invasive and easy method of collection. As this biosensor–app system is intended to be used in clinical practice for the assessment of patient’s saliva samples, artificial saliva was used to mimic the matrix. Artificial saliva is more complex than a simple paracetamol solution; however, human saliva is even more complex, and may introduce interferences from various endogenous substances. But before using human saliva samples, first, it was necessary to develop and adapt both methods in the laboratory to be able to measure concentrations with the mobile application. Drug concentration measurements were performed using a mobile application, MediMeter, developed specifically for this study. The same app could be successfully used for both the detection and quantification of the drug, measured using both the colourimetric and electrochemical methods.

Paracetamol was selected as a model drug to evaluate the biosensor–app system. This drug is commonly used to alleviate pain and reduce fever, and is generally considered safe when administered within recommended therapeutic doses [46,47]. However, the widespread availability of this medication has led to a concerning prevalence of paracetamol overdosing cases. Such instances can have detrimental consequences, potentially resulting in severe adverse effects, including but not limited to hepatotoxicity, liver failure, renal failure, and, in the worst cases, even fatalities [46,47].

The therapeutic dose of paracetamol ranges from 500 to 1000 mg for adults, with the maximum recommended dose being 3 g per day [44] and its hepatic toxicity initiating at plasma concentrations of 100 mg/L [48,49]. In a study involving patients who self-exposed to a potentially toxic paracetamol dose (mean reported dose = 10.3 g), an electrochemical method was employed to establish the correlation between plasma and saliva paracetamol levels [50]. The concentrations at which paracetamol induced damage were determined to be 116 and 62 mg/L in plasma and saliva, respectively. It is essential to note that this study had limitations, being relatively small in scale, with the majority of paracetamol levels falling within the lower range. Additionally, only three plasma levels exceeded 100 mg/L, and none surpassed 150 mg/L. As a result, to conclusively validate the correlation between plasma and saliva paracetamol concentrations above 150 mg/L, a more extensive study with a broader range of concentrations would be necessary.

Given the gravity of the risks associated with paracetamol misuse or accidental overdose, the urgent need for a quick and easy method to detect and quantify paracetamol concentrations cannot be overstated [51,52]. Such a diagnostic tool would not only aid in timely medical interventions, but would also significantly impact the clinical landscape by enhancing patient safety, enabling swift and precise dosing adjustments, and ultimately contributing to more effective healthcare delivery [53].

**Figure 1:**
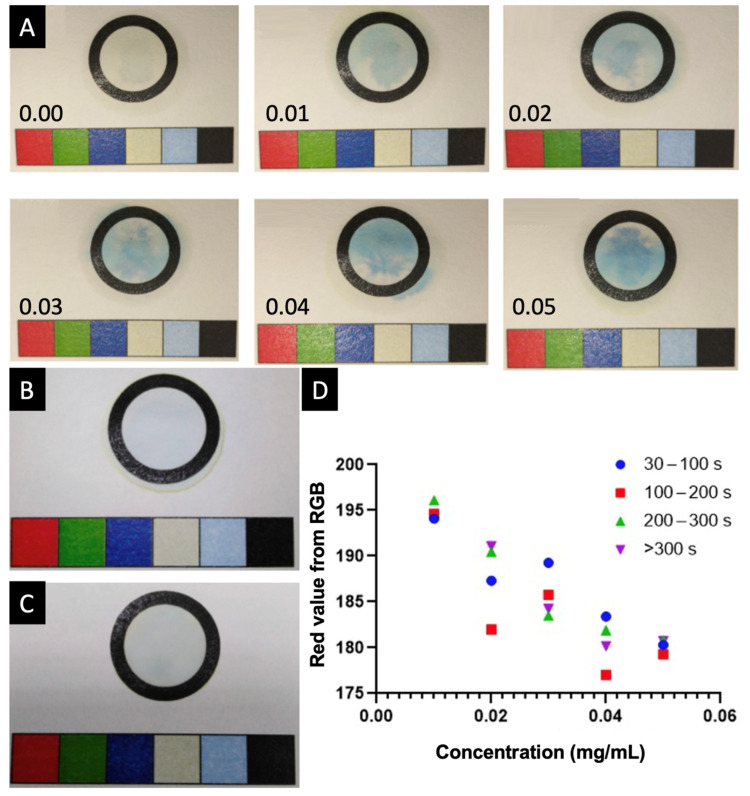
(**A**) Photographs of Prussian Blue using different paracetamol concentrations, ranging from 0.00 to 0.05 mg/mL. Photographs of the template, taken (**B**) with and (**C**) without flash. (**D**) Red colour adjustment using known paracetamol concentrations at different flash exposure times.

In this study, the suitability of the colourimetric and electrochemical techniques was explored for the development of a biosensors–app system. Primarily, the colourimetric method was based on the Prussian Blue reaction. In the context of the colourimetric reaction, the development of a paper template was imperative to constrain the reaction space effectively. Various experiments were conducted to determine the optimal parameters for the reaction space, paper type, printing equipment, and drying methods. Initially, an attempt was made to set the reaction space by subjecting the paper to a 15 min heat treatment at 160 °C in an oven [54]. However, this heating process caused alterations in the paper’s composition, leading to unintended reactions with the Prussian Blue reagents. Consequently, templates were created without subjecting the paper to heat treatment, and the drying process was only carried out at room temperature, as depicted in Figure 1B. Accurate adjustments were also made to the reagents’ volumes without direct contact with the reaction circle.

Various types of paper were tested to identify the one causing minimal interference. This included DP135150 filter paper, 1FCSTA 130 filter paper, coffee filters, Xerox Performer A4 80 g/m^2^, and Whatman Cellulose Chromatography paper grade 1. Ultimately, the decision was made to use Whatman Chromatography paper grade 1, as it exhibited minimal interference compared to other filter papers and helped constrain the reaction space (Figure 1C). This paper is a smooth-surfaced, 0.18 mm-thick cellulose-based material, exhibiting a linear water flow rate of 130 mm/30 min. Of the evaluated printers (PIXMA G3501 (Canon Inc., Tokyo, Japan), Xpress 2020W (Samsung Electronics Co., Ltd., Suwon, Republic of Korea), OfficeJet 8012 (Hewlett-Packard Inc., Palo Alto, CA, USA) and iRC2380i (Canon Inc., Tokyo, Japan)), the Canon iRC2380i printer provided the best printing resolution on the different papers.

Attention was paid to maintaining consistency throughout the procedure. Thus, photographs were captured in identical environmental conditions (Figure 1A), ensuring uniform lighting and flash settings, with image acquisition taking place for up to ~5 min. The establishment of the therapeutic range involved a rigorous procedure of fine-tuning using linear regression, yielding a correlation coefficient of 0.9388. It was imperative to determine whether photographs should be taken with or without flash. Photographs captured without the use of flash resulted in uneven shadows (Figure 1C), hindering precise colour calibration. As a result, the decision was made to employ flash photography (Figure 1B).

To ensure a consistent timeframe, photographs of each circular sample were taken every 30 s over a span of 5.5 min. This process involved utilising the MediMeter app within a researcher’s profile utilising the new calibration mode. The images were then transmitted to a server for the colour information extraction, followed by logarithmic adjustments that led to the formation of four distinct groups: 30 to 100 s, 100 to 200 s, 200 to 300 s, and more than 300 s (Figure 1D). The optimal adjustment was found to be beyond 300 s due to the greater variation in blue intensity observed within this timeframe. Consequently, photographs were consistently taken between 300 and 315 s.

The app offered the capacity to manage the data output of each sample efficiently. It also provided the advantage of being linked to a server where all images, exposure times, and input data were securely stored. In the user profile, the app facilitated the storage of measurements. Its use greatly aided in the correction of photograph colours through the calibration bar, ensuring that changes in lighting conditions or the surface on which the photograph was taken did not affect the results. Nevertheless, employing flash for photography necessitated precise smartphone placement; otherwise, the flash produced unwanted reflections on the calibration bar (Figure S3), obstructing the app’s ability to recognise it. Fortunately, this issue could be assessed before uploading the photograph, since the app displays the image for review beforehand. An alternative solution considered for managing lighting conditions involved adjusting the exposure settings.

An inherent limitation of the Prussian Blue reaction was that any potential reductive substance, including other drugs, would react with ferric sulphate (III) and generate Prussian Blue. Consequently, any substance present in the patient’s saliva with the potential for oxidation could alter the intensity of Prussian Blue, leading to false positive outcomes. This is an important consideration when working with human saliva, as opposed to the artificial saliva examined in this study.

**Figure 2:**
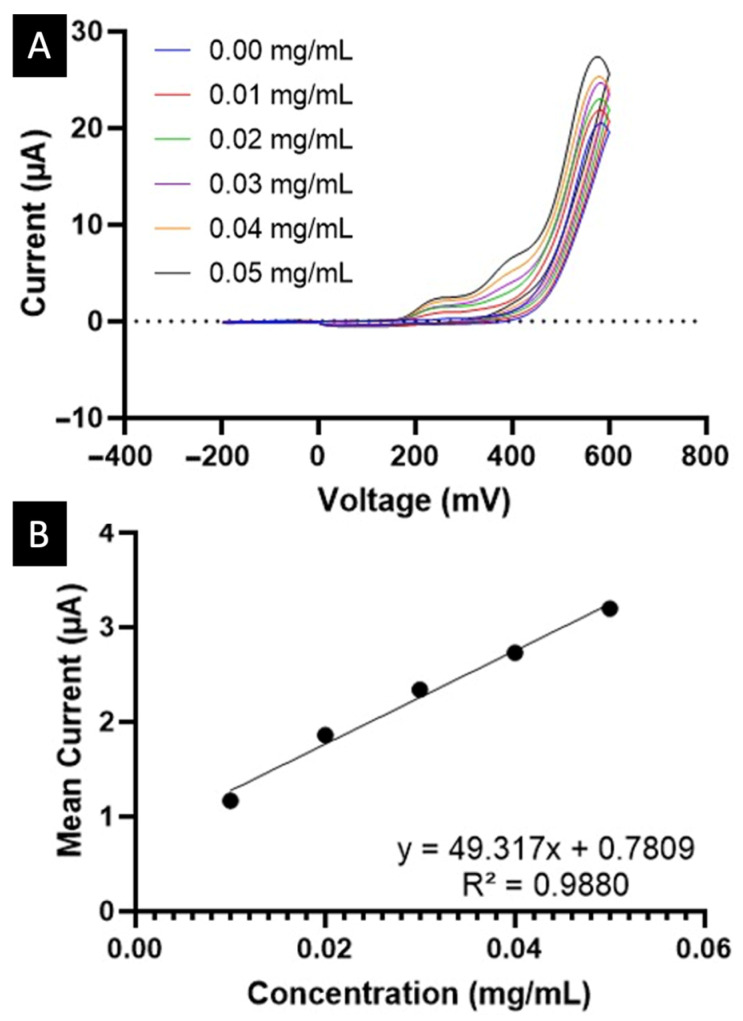
(**A**) Voltammograms of paracetamol samples in artificial saliva at different known concentrations, ranging from 0.00 to 0.05 mg/mL. (**B**) Calibration curve used for paracetamol electrochemical determination at an intensity value of 288 mV.

Paracetamol concentrations were also assessed using an electrochemical approach. Examples of electrochemical devices available on the market include the KickStat [43], JUAMI [55], PSoC-Stat [56], and MiniStat [57] potentiostats. Of these, the KickStat was selected for the electrochemical biosensing combined with the MediMeter app due to its advantages. KickStat combines extended electrochemical capabilities with a compact form factor, measuring a mere 21.6 *×* 20.3 mm. This is crucial for its application in PoC settings, or even for use in a patient’s home. What sets it apart is its cost-effectiveness, with an approximate price tag of just €25 per device, which makes it affordable and accessible for patients [43]. One of its standout features is its operational voltage of 3.3 V, coupled with a resolution of ~1 mV, a level of performance that stands up favourably even when compared to high-end benchtop potentiostats [43]. Figure 2A illustrates one of the voltammograms obtained for a paracetamol sample in artificial saliva. The peak intensity reached its maximum at 288 mV and the calibration curve exhibited a correlation coefficient of 0.9880.

**Figure 3:**
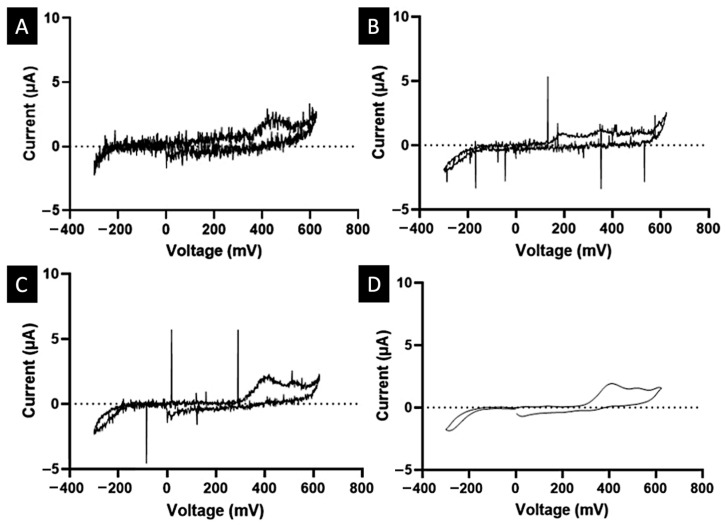
Cyclic voltammetry of 0.05 mg/mL paracetamol in artificial saliva, under the following conditions: (**A**) without buffer and using wires without mesh, (**B**) without buffer and using wires with mesh, (**C**) with 0.1 M phosphate buffer and using wires with mesh, and (**D**) with 0.1 M phosphate buffer and using wires with mesh after smoothing.

Initially, the electrochemical setup involved employing wires without mesh (Figure S2F), resulting in a graphical output resembling that shown in Figure 3A. However, significant improvements were observed when switching to shielding wires (Figure S2A), as seen in Figure 3B. Despite these enhancements, sporadic spikes persisted. To mitigate noise and eliminate spikes, 0.1 M phosphate buffer (pH 6.7) was introduced into the artificial saliva, which reduced the noise but failed to eliminate the spikes (Figure 3C).

To address this, a moving median filter was implemented, employing a window of a specific size that encompassed a set of data points. This filter traversed through the data vector, calculating the median within the window to produce a new “clean” value. This method, by selecting the median from sorted numbers within the window, effectively removed extreme values. To further smooth the resulting graphs, a Gaussian filter was employed. This technique used a moving window to calculate new values, assigning weights to neighbouring elements according to a Gaussian function (Figure 3D).

All these electrochemical method refinements were integrated into the MediMeter app, streamlining future determinations. In each analysis, experimental results are returned, including the graph, raw data, and data that have been processed. After five experiments with the same drug, the developed prototype app generated a new model. Users could access and use the most up-to-date model, with all user measurements securely stored on the server for future reference. When used in a clinical setting, this electrochemical biosensor–app system could possibly show interferences related to the complex content of the human saliva. To resolve this, a new calibration would need to be performed prior to measuring the drug concentration in the patients’ saliva samples (as described in Section 3.2.5). If interferences (noise and spikes) arise, the generated voltametric curves could be refined using different filters to “clean” them. In addition, the MediMeter app will continuously generate new models after carrying out five experiments for a given drug, providing the most up-to-date model, which minimises interferences.

Both techniques successfully enabled the determination of paracetamol concentration in artificial saliva. Notably, the electrochemical method demonstrated greater reliability despite its higher *CV* (Table 1). Only two samples from the therapeutic range fell below the *LOQ*, and all samples exceeded the detection limit, thus indicating that paracetamol concentrations at 0.02 mg/mL or lower cannot be reliably quantified, but all concentration levels can be detected. In contrast, the colourimetric method was able to detect samples at concentrations of 0.04 mg/mL or higher, but all the measured concentrations were outside the limit of quantification. This implies that while the colourimetric approach showed promise in detecting higher concentrations of paracetamol, it may not provide accurate quantification within the specified range. Hence, its performance fell short compared to the electrochemical method, possibly due to the relatively high dispersion of this method (SD = 4.6164 mg/mL). The high dispersion could be attributed to some disturbances in each measurement. The MediMeter app takes a photograph of the sample and the RGB is determined using a scale bar correlating to the drug concentration. Ideally, the photographs should be taken in the same environmental conditions, maintaining the same light intensity, flash settings and distance. However, this may not always be possible, and the presence of small disturbances could translate into variations in each measurement. As a result, the further refinement and optimisation of the colourimetric method may be necessary to enhance its precision and expand its quantification capabilities before testing it with human samples. The tested concentrations (0.01–0.05 mg/mL) in the calibration mode were lower than the toxic concentrations previously determined in human saliva (0.062 mg/mL). This is because the aim is to detect paracetamol concentrations that signal an “alarm” before reaching levels that could cause harm.

The electrochemical method proved to be faster in determining paracetamol concentration, requiring approximately 1 min per sample compared to the colourimetric method, which required roughly 5 min per sample. Even though the colourimetric process proved slower than the electrochemical one, it remains faster than other colourimetric techniques for use in paracetamol detection (e.g., liquid–liquid microextraction combined with digital image colourimetry: 20 min [58]).

Colourimetric and electrochemical biosensors were successfully investigated in previous studies. Compared to prior electrochemical methodologies utilised for paracetamol detection, the method described here exhibits a lower *LOD*, enhancing its sensitivity. For instance, it surpasses existing techniques, including those using a dipyrromethene-Cu(II) monolayers-modified gold electrode (1.2 × 10^−4^ M [59]); the zucchini tissue biosensor (6.9 × 10^−5^ M) [60]; the modified glassy carbon electrode (7.17 × 10^−4^ M) [61]; and the avocado tissue biosensor (8.8 × 10^−5^ M) [62]. Moreover, the developed colourimetric method demonstrates a more sensitive detection limits compared to previously described colourimetric approaches for paracetamol detection (e.g., oxidase-like nanozyme—2.4 μM [63]; silver nanoparticles—0.32 μM [64]), making it more reliable. Furthermore, when it comes to the lowest quantifiable concentrations, the present study exhibits superior precision in measuring paracetamol concentration in comparison to earlier investigations (e.g., modified glassy carbon electrode—2.39 × 10^−3^ M [61]). In terms of *LOD*, the current approaches also outperform other biosensing methods, such as surface-enhanced Raman spectroscopy (SERS; 1.1 × 10^−4^ M) [62]. While the *LOQ* of the SERS approach (7.0 × 10^−4^ M) is better than the colourimetric approach presented herein, the electrochemical system still delivers a more robust performance.

The colourimetric and electrochemical biosensor–mobile app systems developed in this work are intended to be used in TDM. TDM involves quantifying drug concentrations in biological fluids, typically in hospital settings, to optimise efficacy and minimise toxicity, particularly for drugs with a narrow therapeutic index, such as tacrolimus and warfarin. The use of these quick and portable biosensors combined with the MediMeter mobile app, at the PoC, provides a range of advantages compared to the traditional blood or urine sampling methods commonly employed for TDM. Firstly, they offer non-invasive sampling, eliminating the discomfort and inconvenience associated with blood collection and urine sample privacy [65,66]. Moreover, hospital visits are reduced since patients can perform measurements in a few minutes, independently at their homes using the MediMeter app, offering a user-friendly experience while safeguarding sensitive patient information. This approach significantly improves patient comfort and compliance while providing real-time feedback. Furthermore, these biosensors are cost-effective as they reduce the need for specialised laboratory equipment and trained personnel, making healthcare more affordable and accessible [67,68,69].

Another key advantage is the real-time monitoring capability these developed biosensors provide using both methods. Patients and healthcare providers can obtain immediate results in just minutes (ranging from 1 min for the electrochemical method to 5 min for the colourimetric method), enabling swift decision-making regarding medication adjustments or interventions [70]. This real-time feedback loop is particularly valuable for managing conditions that necessitate continuous monitoring [71,72,73]. Furthermore, these biosensors reduce dependence on centralised laboratories, which often lead to delays in obtaining results due to the techniques employed for quantification (HPLC-UV or MS-MS). Testing directly at the PoC is especially crucial in emergencies and remote or resource-limited settings [74,75]. Lately, smartphone-based sensing systems have garnered increased interest due to their user-friendly, semi-automated interface that can be utilised by the general population without the need for specialised training or technical expertise [76,77]. In the future, it is anticipated that a closed-loop digital healthcare system will see the seamless integration of smartphone or wireless biosensing systems [78]. This integration will involve the incorporation of biosensing, wireless data collection and transmittance, remote diagnosis and patient monitoring, and the provision of on-demand outputs tailored to individual needs, including personalised therapies [78,79].

Additionally, saliva sampling, compared to urine, is less affected by factors like fluid intake and diurnal variations [80]. Saliva composition remains relatively stable throughout the day, ensuring consistent and reliable measurements [81,82]. Furthermore, quick biosensors promote patient-centric care by allowing patients to actively engage in their healthcare. They can conveniently monitor their drug concentrations from home or while on the go, enhancing the self-management of chronic conditions [78]. It is important to highlight that the current work was conducted using artificial saliva, and therefore, when tested using human saliva, this biosensor system may not have the same sensitivity. Whilst previous studies have shown that biosensing results using artificial saliva can be correlated to those obtained using human saliva samples [83,84], this is not always the case [85,86]. Thus, the current system needs to be validated using human saliva to ensure similar sensitivity in the resulting practice.

It is important to note that biosensors encompass a diverse range of technologies, and the performance of these sensors can vary significantly based on their specific design and the nature of the drug being analysed [87,88]. Therefore, optimisation is a crucial aspect of biosensor development and deployment [89]. The developed colourimetric and electrochemical biosensors, combined with the MediMeter mobile app, can be seamlessly adapted for monitoring other drugs, particularly those with narrow therapeutic indices. The MediMeter app’s ability to store calibration data for multiple drugs facilitates the creation of a comprehensive drug library. The choice of biosensor type, whether colourimetric, electrochemical, or another variant, will impact its sensitivity, precision, and speed, as demonstrated in this study as well as previous ones [90,91]. Furthermore, different drugs may exhibit distinct behaviours and interactions within the sensor, necessitating tailored optimisation strategies. In this study, it was recognised that optimisation was a continual process, and the findings emphasise the importance of adapting biosensors to suit specific applications and drugs. This adaptability ensures that biosensors can consistently deliver accurate and reliable results across a spectrum of clinical scenarios. As an example, the study revealed that different types of paper significantly influence the performance of the colourimetric biosensing method. Thus, it is imperative to recognise that the choice of paper substrate can impact the accuracy and reliability of the measurements obtained. Consequently, if alternative types or colours of paper were to be considered for use, it is essential to revalidate the method to ensure its continued effectiveness and precision.

This study focused on developing two biosensing techniques, integrated with the MediMeter mobile app, to detect and quantify paracetamol in artificial saliva. The next step would involve evaluating the biosensor–app system using human saliva and comparing the results with conventional hospital methods, such as HPLC. Given its user-friendly interface, the MediMeter app has the potential to enable patients or healthcare professionals to perform measurements at home or at the PoC, reducing the need for hospital visits. Based on the study’s findings, the electrochemical technique appears more suitable for initial clinical testing than the colourimetric method. This is due to the latter’s reliance on the Prussian Blue reaction, which may be affected by oxidisable substances in the patient’s saliva, potentially leading to false-positive results.

## 3. Materials and Methods

### 3.1. Materials

Paracetamol (also known as acetaminophen, USP grade, MW 151.16 g/mol, solubility in water at 37 °C—21.80 g/L) was obtained from Merck KGaA (Sigma-Aldrich, Darmstadt, Germany) Sodium chloride, sodium hydrogen carbonate, di-sodium hydrogen phosphate and potassium thiocyanate were purchased from Merck KGaA (Sigma-Aldrich, Darmstadt, Germany). Potassium chloride and potassium dihydrogen phosphate were provided by ITW Reagents PanReac (Barcelona, Spain). Potassium hexacyanoferrate (ferricyanide) (III) (MW 329.26 g/mol) was purchased from Thermo Scientific Chemicals (Waltham, MA, USA). Ferric sulphate hydrate (MW 399.9 g/mol) was purchased from MP Biomedicals (Santa Ana, CA, USA). Whatman Grade 1 Chr Cellulose Chromatography paper (46 × 57 cm) was procured from GE Healthcare (Chicago, IL, USA). Working electrodes (gold and glassy carbon), the counter electrode (Pt) and the reference electrode (Ag/AgCl) were provided by BASi^®^ Research Products (West Lafayette, IN, USA). Screen-printed electrodes were provided by Metrohm DropSens S.L. (Asturias, Spain). All utilised chemicals and reagents were of analytical grade.

### 3.2. Methods

#### 3.2.1. Artificial Saliva Preparation

Artificial saliva (AFNOR NF standard S90-701) [92] was prepared as previously described in the literature [93,94]. Briefly, 6.7 g of sodium chloride, 1.2 g of potassium chloride, 0.26 g of di-sodium hydrogen phosphate, 0.2 g potassium dihydrogen phosphate, 1.5 g of sodium hydrogen carbonate, and 0.33 g of potassium thiocyanate were dissolved in 1 L of deionised water. Following the dissolution of all the components, the pH was adjusted to 7.0 by adding orthophosphoric acid or sodium hydroxide.

#### 3.2.2. Paracetamol Samples Preparation

A paracetamol stock solution was prepared by dissolving 25 mg of paracetamol in 500 mL of artificial saliva, yielding a final concentration of 0.05 mg/mL. Subsequently, a series of dilutions were carried out to prepare paracetamol solutions at concentrations of 0.04, 0.03, 0.02, and 0.01 mg/mL. These dilutions were prepared by mixing the stock solution with artificial saliva in 25 mL volumetric flasks.

#### 3.2.3. Colour Reagent Preparation

The reagents were formulated by combining equal volumes of (a) 15 mM ferric sulphate (III) hydrate and (b) 15 mM potassium hexacyanoferrate (III). The preparation of these solutions involved dissolving 0.6 g of ferric sulphate hydrate in 100 mL of deionised water to create the ferric sulphate solution, and separately dissolving 0.494 g of potassium hexacyanoferrate (III) in 100 mL of deionised water to create the potassium hexacyanoferrate (III) solution.

#### 3.2.4. Mobile Application Development

**Figure 4:**
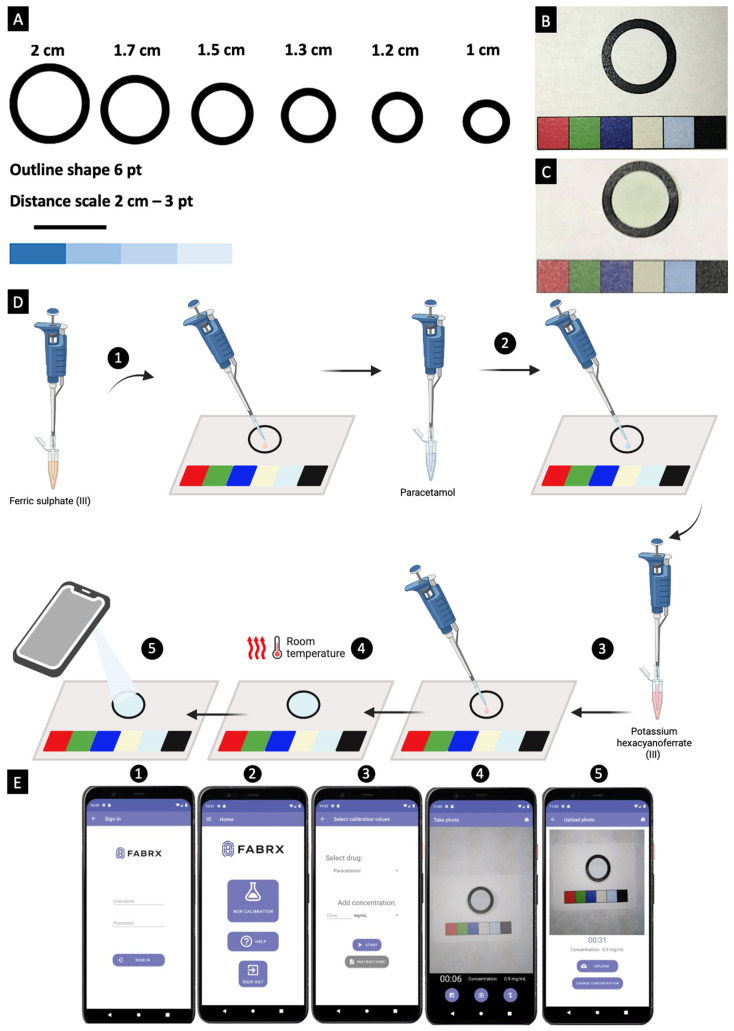
(**A**) Design of the preliminary template. Photographs of the (**B**) final template and (**C**) a colourimetric template with the Prussian Blue reagents on it. (**D**) Graphical illustration of the colourimetric method’s procedure. Steps include: (1) addition of ferric sulphate (III) onto the test paper, (2) addition of the drug, (3) addition of potassium hexacyanoferrate (III), (4) air-drying the paper to allow Prussian Blue to form in the reaction ring, and (5) scanning the paper using a smartphone and uploading the image onto the MediMeter app for analysis. (**E**) Images of the different pages of the colourimetric quantification experimental MediMeter app, including (1) the login page, (2) main menu to start a new calibration, (3) calibration mode to select the drug and the concentrations before taking pictures, (4) photo mode, and (5) image upload or repetition options.

**Figure 5:**
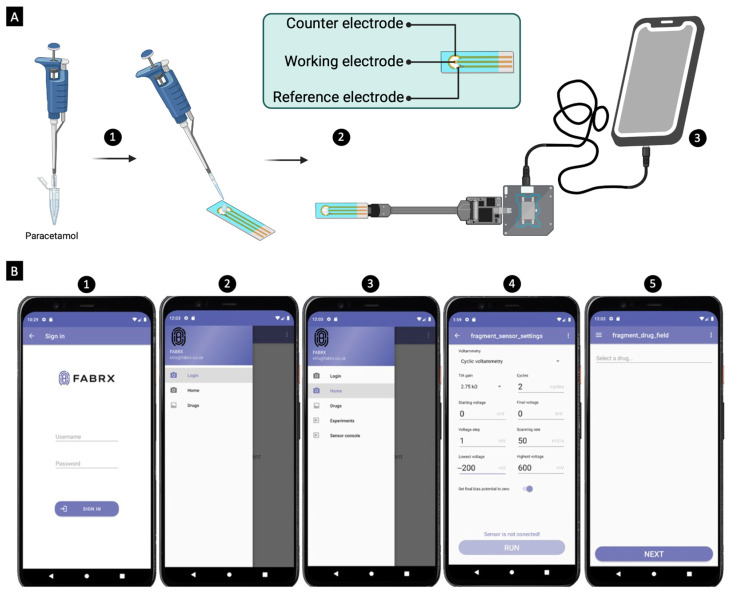
(**A**) Graphical illustration of the electrochemical method’s procedure. Steps include (1) addition of the drug onto the screen-printed electrodes, (2) connecting the electrodes to a potentiostat, which generates voltammograms of the paracetamol samples, and (3) the transmission of the voltammograms to the MediMeter app for analysis. (**B**) Visual representations of the electrochemical MediMeter app, demonstrating essential aspects such as (1) the login page, (2) user’s menu for navigation, (3) main menu offering specialised functions, (4) voltammetry screen enabling researchers to fine-tune assay parameters, and (5) drug selection menu.

The MediMeter mobile application (FABRX AI, O Saviñao, Spain) was designed specifically for the detection and quantification of drug concentrations using both the colourimetric and electrochemical techniques. Upon launching the app, a login screen appears, requiring a username and password. This ensures that each patient or healthcare professional has individualised access, safeguarding their privacy. Screenshots illustrating the use of the app for both methods are provided in Figure 4E and Figure 5B. As part of the procedure (further detailed in the following sections), the app first conducts a calibration. Once calibrated, the app is used to measure samples with known concentrations of the drug (ranging from 0.01 to 0.05 mg/mL), and it reports the corresponding concentration value. All measurement data are securely stored on a server, with encrypted data ensuring patient privacy. The system’s design ensures that access to the stored data is strictly restricted, with only authorised individuals able to view it, provided they have the correct ID and password.

#### 3.2.5. Colourimetric Calibration and Quantification

The colourimetric method for quantifying paracetamol involved a sequential process of two chemical reactions. Initially, the drug underwent a redox reaction with ferric sulphate (III), resulting in the reduction of iron from its ferric (III) state to ferrous (II). Subsequently, the ferrous (II) ions react with potassium hexacyanoferrate (III), ultimately yielding ferric ferrocyanide, denoted as Fe4[Fe(CN)6]3 or Prussian Blue (Figure S1) [95]. Notably, the intensity of the resulting colour, in this case, blue, directly corresponds to the drug quantity present, with a higher drug concentration yielding a more pronounced blue colouration.

Paper templates were printed on Whatman Cellulose Chromatography paper grade 1 using a Canon iRC2380i printer (Canon Inc., Tokyo, Japan) (Figure 4A–C), although several designs and papers were used in preliminary tests. The final template included a 1.5 cm diameter circle as the reaction space and a calibration bar with a specific colour pattern used for camera calibration to detect the light conditions and the distance to the paper. The colourimetric procedure entailed micropipetting 7 μL of ferric sulphate (III) and dispensing it in the reaction space of the test paper (Figure 4D). This was followed by the addition of 7 μL of paracetamol solution. Subsequently, 7 μL of potassium hexacyanoferrate (III) was introduced, forming Prussian Blue in the reaction ring. This process was carried out for six different paracetamol concentrations, ranging from 0 to 0.05 mg/mL, and each concentration was replicated five times, resulting in a total of 30 samples.

Photographs of each circle were captured every 30 s over a duration of 5.5 min using an OPPO A54 smartphone (OPPO, Dongguan, China) running Android 11, with the MediMeter app (FABRX AI, O Saviñao, Spain; Figure 4E), featuring a user profile. The app automatically helps the user determine the image capturing distance, ensuring reproducibility between measurements. These images were transmitted to a server for colour information extraction, and a linear adjustment was applied. Following the image capture, Python’s OpenCV (Version 4.7.0.68, Python Software Foundation, Wilmington, DE, USA) was utilised to identify the image contours, including those of the calibration bar and the circle. Once the contours were detected, the calibration bar was divided into six equal segments, and the average colour was obtained from each segment. For the circle, K-means clustering was employed to identify the bluest region, characterised by a higher B (blue) component compared to the R (red) and G (green) components. These values were obtained for each image and were employed as feature vectors for constructing a model. Linear regression was then applied to predict paracetamol’s concentration. Once the model was created, the coefficients, intercept, model name, device, and the corresponding medication were stored in the database.

To validate the calibration, samples with known concentrations (i.e., 0.01, 0.02, 0.03, 0.04, and 0.05 mg/mL) of paracetamol were prepared, and paracetamol’s concentration was quantified using the app within the user profile. When a user selects the drug to be monitored, the app automatically downloads the corresponding model. Upon taking a photograph, the RGB values of the circle are acquired, and using the stored coefficients and intercept, the drug concentration is calculated and displayed to the user.

#### 3.2.6. Electrochemical Calibration and Quantification

Cyclic voltammetry assays were conducted using a KickStat potentiostat (PCBWay Hangzhou, China; Figure S2B), connected to a three-electrode system (Figure 5A). Initially, the system featured (a) a gold working electrode (1.6 mm in diameter; Figure S2C), (b) a platinum (Pt) wire counter electrode (Figure S2D), and (c) an Ag/AgCl reference electrode (saturated KCl; Figure S2E). The entire setup was controlled through the Arduino Software (Version 1.8.12, Arduino, Somerville, MA, USA). The experiments were carried out at pH 4.6 (utilising an acetate buffer) and pH 6.7 (utilising a phosphate buffer), at a constant temperature of 25 °C. The voltametric curves were recorded using a cell configuration that contained a 50 mL solution of paracetamol with concentrations ranging from 0.01 to 0.05 mg/mL.

Later on, screen-printed electrodes (Figure S2G) replaced the three-electrode system. They were connected to the potentiostat using wires (Figure S2H) and were used for the calibration process. Subsequently, drops of samples dissolved in artificial saliva mixed with phosphate buffer were added. Thereafter, the same software configuration as with the colourimetric approach was applied, with the analysis carried out at 25 °C (Figure 5B).

To calibrate paracetamol (without buffer) at 25 °C, an OPPO A54 smartphone (OPPO, Dongguan, China) running Android 11 was employed, utilising the MediMeter app (FABRX AI, Spain). The voltametric curves were generated with a cell configuration involving 25 mL of paracetamol solution, spanning concentrations from 0.01 to 0.05 mg/mL. The potential window extended from −0.200 V to +0.600 V, with a scan rate of 50 mV/s and a step potential of 1 mV.

The application translates the form field values into commands. The microcontroller firmware, which awaits instructions, initiates the voltammetry technique according to the specified parameters. The application receives the output from the Arduino board through the serial port. Upon completion of the experiment, the experimental object is stored, allowing for the assignment of an associating identifier to the remaining fields, and the collected data are processed. The application aggregates the board’s output into a comma-separated values (CSV) file, in which graphics for each cycle are generated, cleaned, and smoothed. This new file is then stored on the server. The app consults the database to determine how many experiments were associated with the particular drug. If there are five or more experiments, a new predictive model construction process is triggered to calculate a fresh regression line, which is subsequently stored in the database.

To validate the calibration, samples of known concentration (i.e., 0.01, 0.02, 0.03, 0.04, and 0.05 mg/mL) were prepared, and the paracetamol concentration was quantified using the app within a specific profile created for this work.

#### 3.2.7. Statistical Analyses

To determine and compare the accuracy of both techniques, parameters such as the limit of quantification (*LOQ*) and limit of detection (*LOD*) were considered to understand their capacities for measuring low-concentration drug levels and ensuring the quality of data obtained. They were calculated using Equation (1) and Equation (2), respectively, as follows:

(1) $$LOQ=\frac{10S_{y}}{S}$$

(2) $$LOD=\frac{3.3S_{y}}{S}$$

where $S_{y}$ is the standard deviation of the response and S is the slope of the calibration curve. The expansion factors 3.3 and 10 were based on a confidence level of 95% [96]. $S_{y}$ was calculated using Equation (3), as follows:

(3) $$S_{y}=\sqrt{\frac{\sum_{i=0}^{n}{\left(y_{i}-\bar{y}\right)}^{2}}{n-1}}$$

where $y_{i}$ represents each individual data point, $\bar{y}$ is the mean of the response variable, and n is the number of data points.

The coefficient of variation (*CV*), expressed as the ratio of the standard deviation to the mean, was used to assess the extent of variation in measured concentrations relative to the calibrated values. To calculate the *%CV*, Equation (4) was used:

(4) $$\%CV=\frac{\sigma }{\mu }\times 100\%$$

## 4. Conclusions

In this study, an exploration of two distinct methodologies, specifically colourimetric and electrochemical techniques, along with the MediMeter mobile app, was conducted to assess the effectiveness of a biosensor–app system in determining paracetamol concentration within artificial saliva. The study involved a systematic examination of various optimisations and methodological enhancements, wherein the judicious choice of materials and conditions enabled the minimisation of external interferences, thereby ensuring that the measurements obtained accurately reflected paracetamol concentration. Significantly, the study outcomes highlighted the proficiency of both methods in effectively quantifying paracetamol concentrations, with the electrochemical approach notably surpassing the colourimetric method in terms of precision and speed.

This research emphasises the substantial potential inherent in both techniques for drug concentration determination, and the findings can be applied to other drug candidates for TDM. This potential opens doors to a plethora of applications across the pharmaceutical and healthcare sectors, including different drugs’ or biomarkers’ real-time determination. The choice between these methodologies may ultimately pivot on the specific requisites of a given application or drug. Considerations such as reliability and efficiency emerge as crucial factors in making this determination. This innovative platform empowers individuals to remotely monitor drug concentrations with unparalleled convenience, obviating the need for invasive methods and hospital visits, which typically entail the involvement of healthcare professionals and extended timeframes. As the focus turns toward the future, abundant opportunities beckon for further research endeavours aimed at translating these methodologies into pragmatic, real-world clinical applications. Through these efforts, the potential exists to redefine the landscape of TDM, ushering in an era distinguished by heightened precision, accessibility, and patient-centric care.

## Figures and Tables

**Table 1:** A comparative summary of paracetamol concentration determination between colourimetric and electrochemical techniques.

	Colourimetric	Electrochemical
*LOD* (mg/mL)	0.0379	0.0070
*LOQ* (mg/mL)	0.1148	0.0211
$S_{y}$ (mg/mL)	4.6164	0.1041
*CV* (%)	3.8544	18.733
Detection time (min)	~5	~1

## Data Availability

Data will be made available on request.

## Supplementary Materials

The following supporting information can be downloaded at: https://www.mdpi.com/article/10.3390/bios15030163/s1, Figure S1: Chemical reaction to form Prussian Blue; Figure S2: Materials used for electrochemical quantification; Figure S3: Example of an image featuring reflections.
