# Supplementary material for: Smartphone Biosensors for Non-Invasive Drug Monitoring in Saliva
Source: Biosensors (Basel). 2025 Mar 4;15(3):163. doi: 10.3390/bios15030163 (PMC11940247; doi:10.3390/bios15030163)
Supplement: Supplementary file 1 [file biosensors-15-00163-s001.zip › biosensors-3377339-supplementary.pdf]

Supplementary Material

Smartphone Biosensors for Non-Invasive Drug Monitoring in Saliva

Atheer Awad [1,2,†], Lucía Rodríguez-Pombo [3,†], Paula Esteiro Simón [3], André Campos Álvarez [4,5], Carmen Alvarez-Lorenzo [3], Abdul W. Basit [2,4,5,*] and Alvaro Goyanes [2,3,4,5,*]

[1] Department of Clinical, Pharmaceutical and Biological Sciences, University of Hertfordshire, College Lane, Hatfield AL10 9AB, UK
[2] Department of Pharmaceutics, UCL School of Pharmacy, University College London, 29-39 Brunswick Square, London WC1N 1AX, UK
[3] Departamento de Farmacología, Farmacia y Tecnología Farmacéutica, I+D Farma (GI-1645), Facultad de Farmacia, Instituto de Materiales (iMATUS) and Health Research Institute of Santiago de Compostela (IDIS), Universidade de Santiago de Compostela, 15782 Santiago de Compostela, Spain
[4] FABRX Ltd., Henwood House, Henwood, Ashford TN24 8DH, UK
[5] FABRX Artificial Intelligence, Carretera de Escairón, 14, Currelos, 27543 O Saviñao, Spain
* Correspondence: a.basit@ucl.ac.uk (A.W.B.); a.goyanes@fabrx.co.uk (A.G.); Tel.: +44-(0)-2039872769 (A.W.B.); +44-(0)74-5488-7793 (A.G.).
† These authors contributed equally to this work.

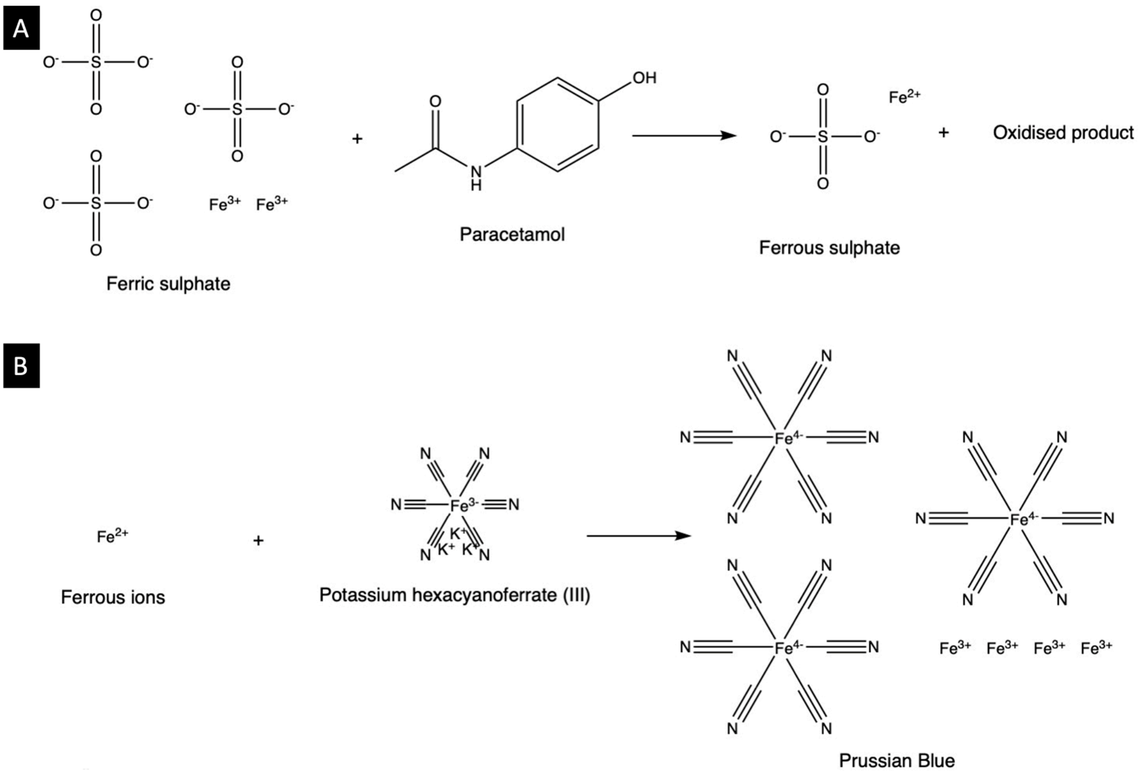

Figure S1. Chemical reaction of (A) paracetamol with ferric sulphate (III), and (B) ferrous ions with potassium hexacyanoferrate (III) to form Prussian Blue.

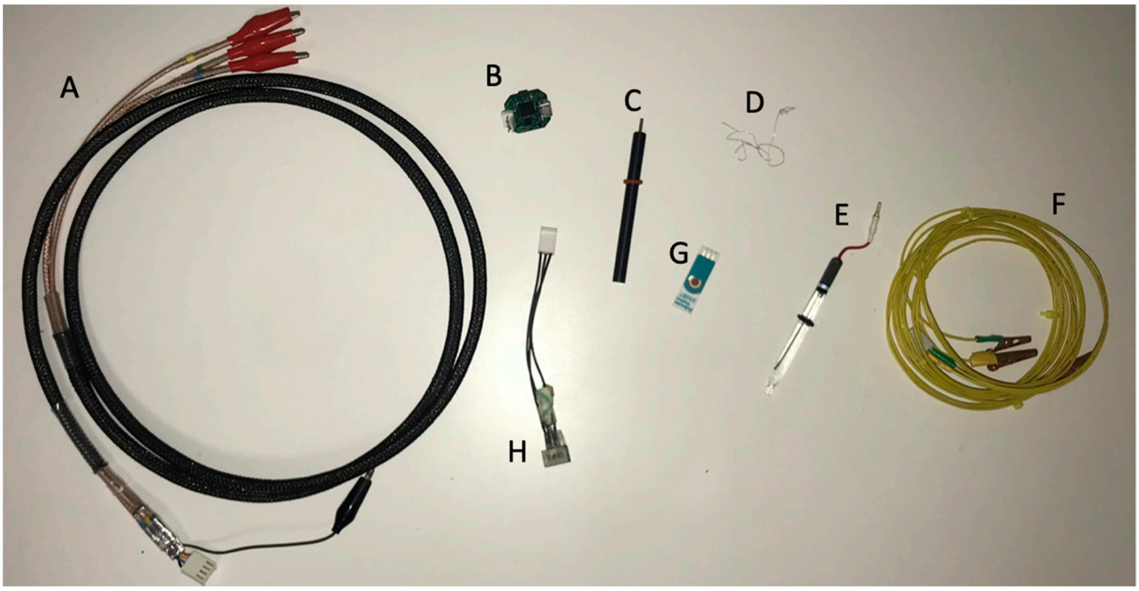

Figure S2. Materials used for electrochemical quantification, including: (A) wires with mesh, (B) a KickStat potentiostat, (C) gold working electrode, (D) platinum counter electrode, (E) Ag/AgCl (saturated KCl) reference electrode, (F) wires without mesh, (G) screen-printed electrodes, and (H) wires used to connect screen-printed electrodes.

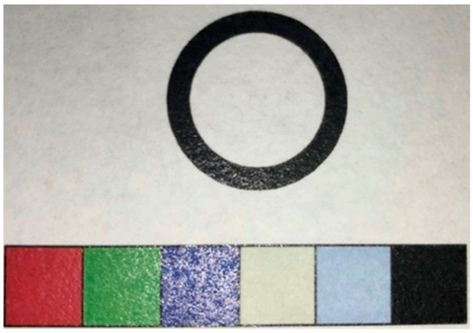

Figure S3. Example of an image featuring reflections on the calibration bar due to improper alignment during smartphone image capture, in conjunction with flash usage.
